# Supplementary figures and images for: Comparative Metabolomic Responses of Three Rhododendron Cultivars to the Azalea Lace Bug (Stephanitis pyrioides)
Source: Plants (Basel). 2024 Sep 13;13(18):2569. doi: 10.3390/plants13182569 (PMC11434956; doi:10.3390/plants13182569)

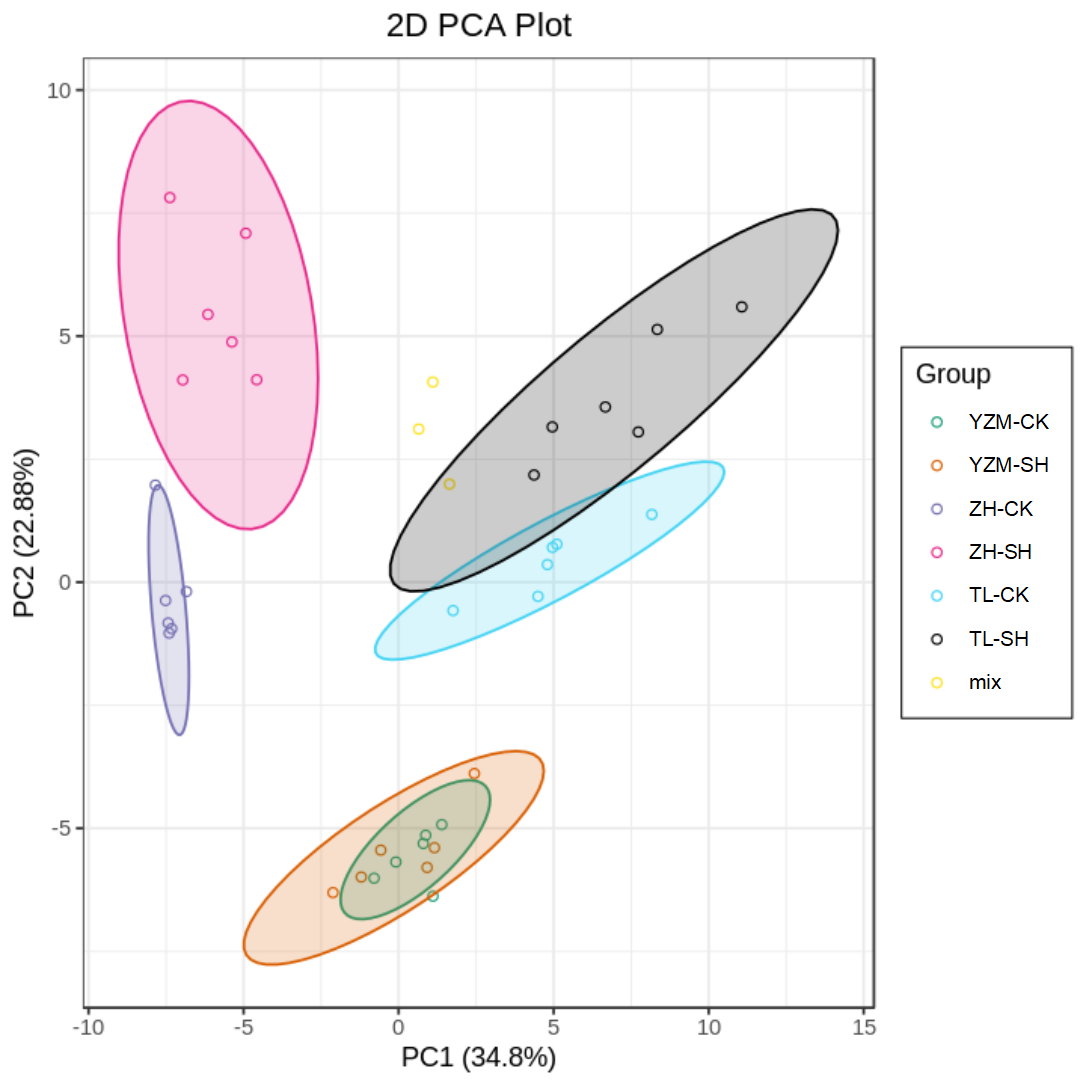

Supplement: Supplementary file 1 [file plants-13-02569-s001.zip › Figure S1 PCA Analysis of Total Samples in GC-MS.png]

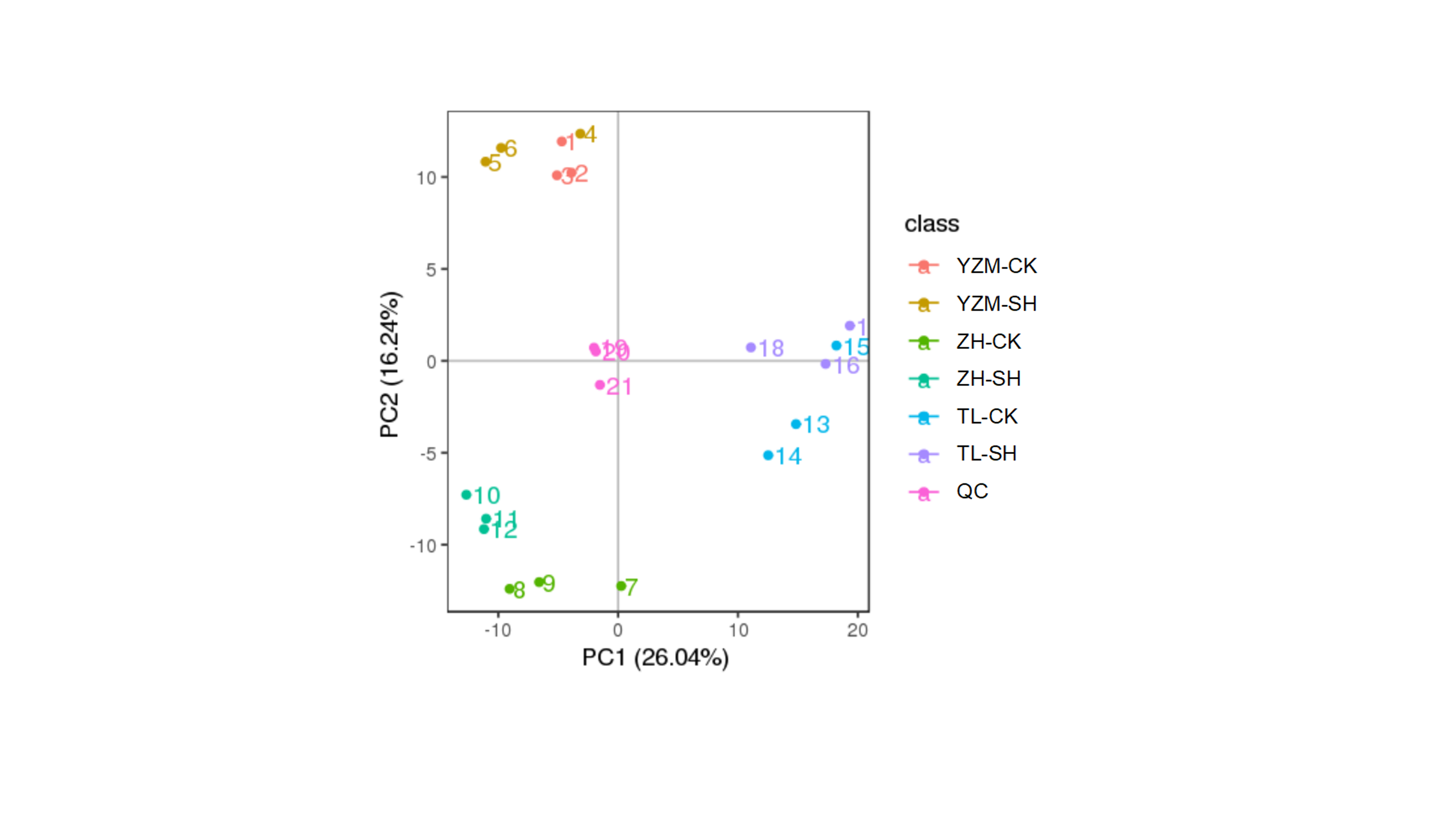

Supplement: Supplementary file 1 [file plants-13-02569-s001.zip › Figure S2 PCA Analysis of Total Samples in LC-MS.png]

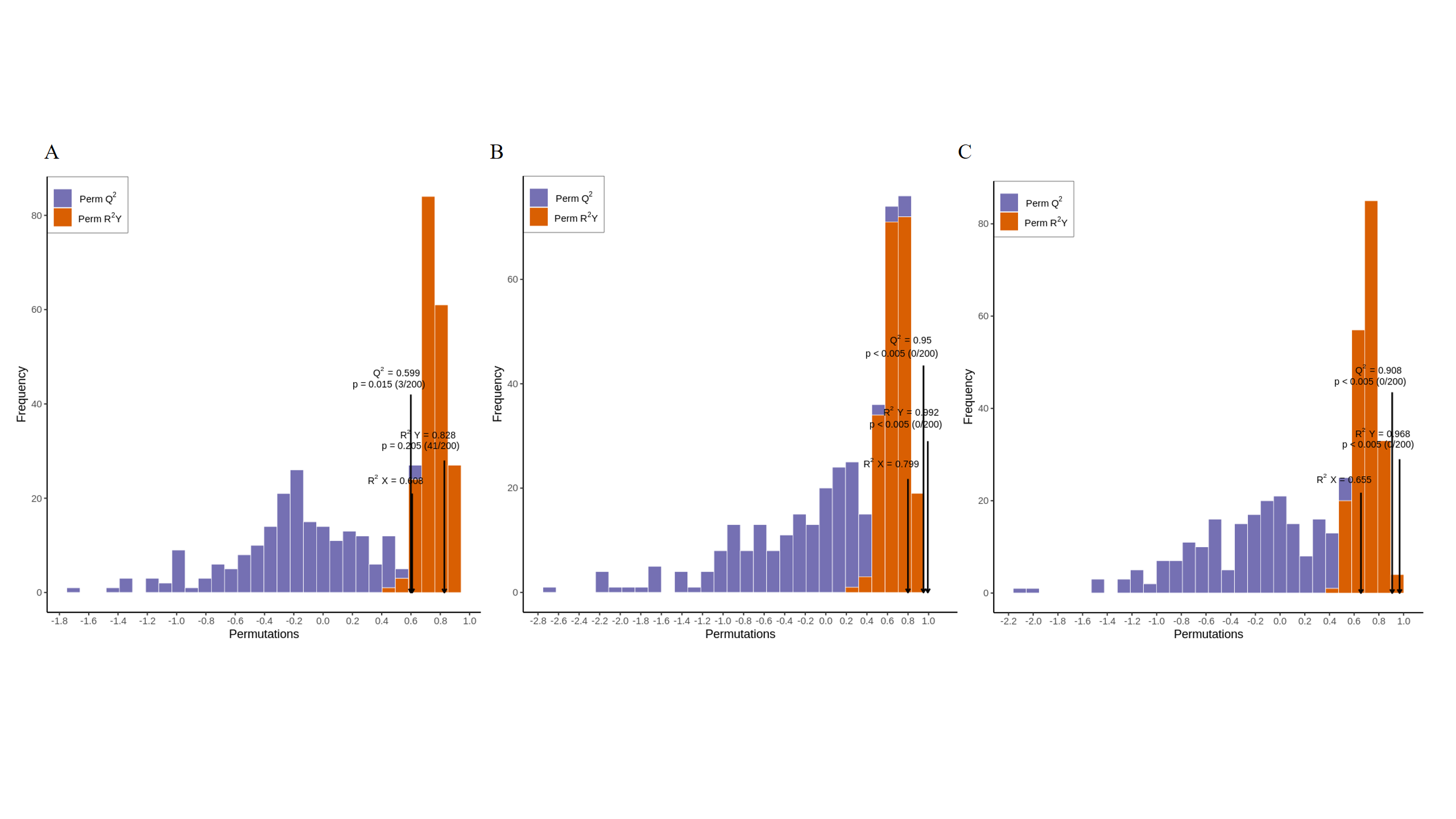

Supplement: Supplementary file 1 [file plants-13-02569-s001.zip › Figure S3 OPLS-DA model validation diagram in GC-MS..png]

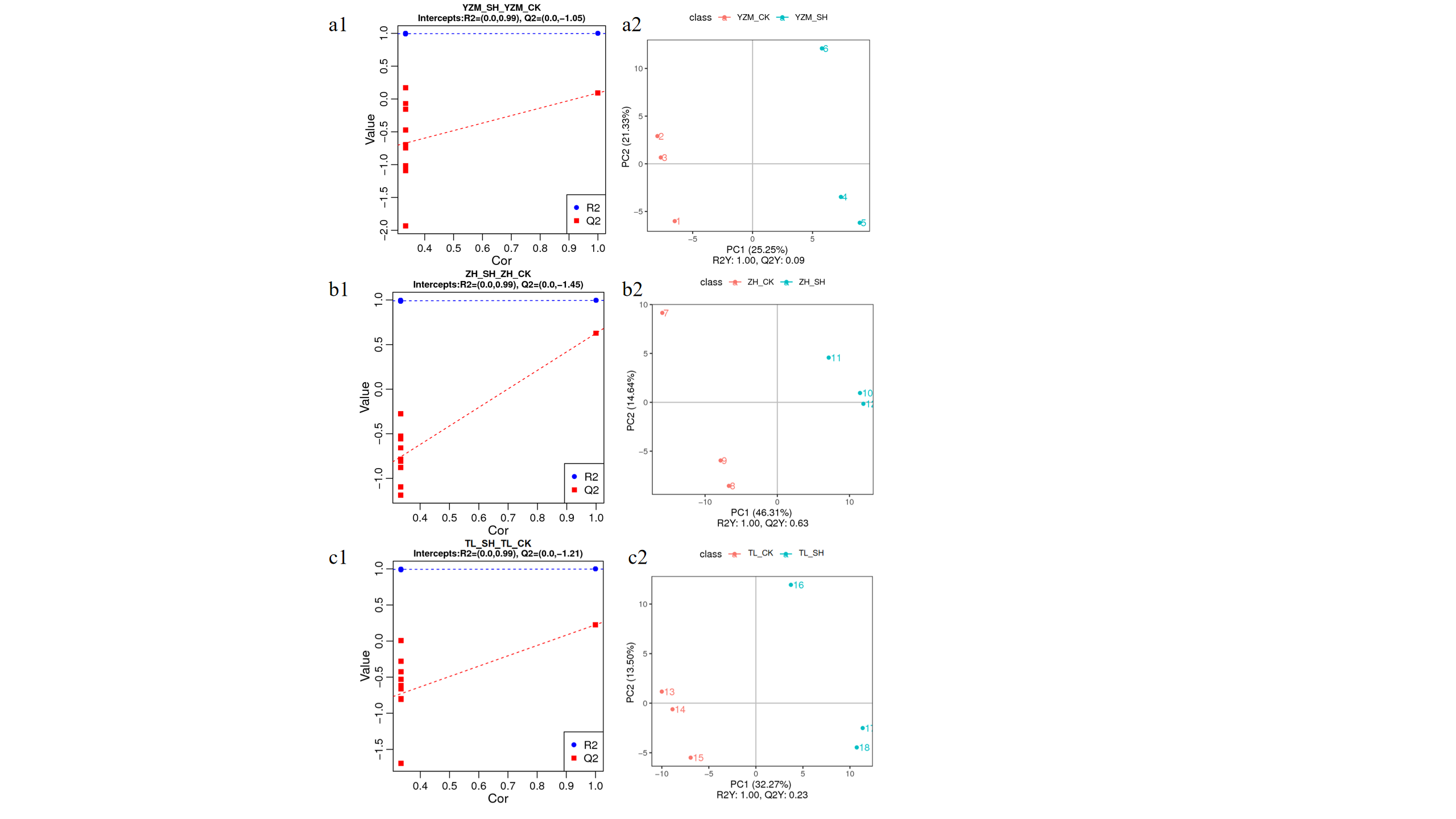

Supplement: Supplementary file 1 [file plants-13-02569-s001.zip › Figure S4 PLS-DA scatter plot and sorting validation plot in LC-MS.png]
